# To Overcome Psychiatric Patients’ Mind–Brain Dualism, Reifying the Mind Won’t Help

**DOI:** 10.3389/fpsyt.2020.00605

**Published:** 2020-06-30

**Authors:** Stephan Schleim

**Affiliations:** Theory and History of Psychology, Heymans Institute for Psychological Research, Faculty of Behavioral and Social Sciences, University of Groningen, Groningen, Netherlands

**Keywords:** dualism, psychiatric disorders, reductionism, biopsychosocial model, mechanistic explanations

## Introduction

Glannon (1) summarized recent research in Biological Psychiatry and discussed possible ethical implications of mind–brain dualism in psychiatry in this journal. He particularly addressed the risk that patients might disregard neuroscientific discoveries and subsequently neglect effective biological treatments for psychiatric disorders. In this opinion article, I want to emphasize how some philosophers and scientists still use dualistic language, leading researchers and, to a lesser degree, clinicians into unnecessary and possibly even confused debates on mind–body reductionism; I also briefly address empirical data suggesting that, in contrast to Glannon, patients at large presently don’t seem to eschew biological treatments.

## From Soul-Body to Mind–Brain Dualism

René Descartes distinguished a *thinking* substance (soul) and *extended* substance (body) and hypothesized, based on his physiological studies, that both interact primarily in humans’ pineal gland (2, 3). Already his contemporaries criticized the lacking explanation of the *mechanism of interaction* between brain/body and soul, as documented, for example, in the letters between Descartes and Princess Elisabeth of the Palatinate (4). Even centuries later, philosophers and neuroscientists have pointed out that the *language use* of many scholars remains problematically dualistic, even when they believe to have long overcome mind–brain dualism (5).

Glannon favors a biopsychosocial stance on psychiatric disorders, acknowledging social, psychological, and neural factors. But he frequently mentions “mind–brain interaction”, though, without explaining this concept. This reiterates Descartes’ unsolved problem. Philosophy of Mind has found no clear answer to the question what “the mind” is. One of the major schools of psychology, Behaviorism, actually found the whole concept suspicious and in need of replacement by more scientific terms (6); a similar idea was later formulated by philosophers as Eliminative Materialism (7). Speaking of “the mind” as if it were a thing interacting with the brain/body (1, 8) is thus by no means trivial.

## Descriptive Levels

Instead, I propose to speak of *processes* which are *psychological* processes if and only if they fall under a psychological description. Similar to Dennett’s *Intentional Stance* (9), this is a pragmatical proposal that requires no strict commitment about whether psychological descriptions will ever be reducible to neuroscientific (or even more basic: physical) descriptions. This is also reflected in psychological experimentation—as well as clinical or cognitive neuroscience inasmuch as they make use of psychological concepts—where researchers *operationalize* psychological concepts to explain people’s experience and behavior without necessarily placing them “in the mind” or reducing them to the brain. Reductions are much less common even in the natural sciences than often assumed in that debate, anyway: Biology and chemistry, for example, are obviously necessary, because there are processes in the world (*e.g.* life) which cannot be described in purely physical language. And it is also not clear what the final, most basic level of a physical description should be (10, 11). That of energy, information, or yet something different? Thus, perhaps not even all physical descriptions might be reducible to the most basic physical level of description, whatever that may be.

The sciences, including human and social sciences like psychology, sociology, and economics, continue to develop in a pluralistic, non-reductive way, informing each other in many respects. And a much more promising alternative to reductive explanations are *mechanistic* explanations (12, 13). They *integrate* different levels of description, instead of replacing them. I will use an example to briefly explain what this could mean for psychiatric disorders.

## An Example

Imagine Karla hearing that her spouse and children died in a traffic accident. Although that accident involved all kinds of physical and biological processes, described as changes of energy matter, molecules, tissues, and so on, they cannot express the significance for Karla that her beloved ones passed away so suddenly. But we need not assume an independent, reified^1^ “mind” to state that at the moment she understands (psychology) the message, all kinds of processes will occur in her: Karla might first react with denial (psychology), a severe stress response might happen in her body (physiology), electric activity and molecules may change in her nervous system (neurophysiology) and likely also affect some neural structures permanently (neuroanatomy).

As time proceeds, Karla probably experiences grief and perhaps even depression; serious life events are indeed the strongest known risk factors for distress and Major Depressive Disorder (MDD) (14, 15). Again, this will also involve processes on different levels, as described before, including neuroscientific levels. Glannon actually summarizes much neuroscientific research consistent with such multi-level descriptions. We may ask what is *specific* about hearing that one’s loved ones died which is causing all these effects; but this does not require us to posit a “mind” entity mysteriously interacting with the brain. Instead, a mechanistic explanation can integrate all levels of description (16), also in line with the biopsychosocial model (17, 18). On the psychological level of description, philosophical concepts like *intentional* (*e.g.* what a thought is about) or *phenomenal* content (*e.g.* what it feels like) that are considered as unique features of the mental domain can play a role, too (19).

This pluralistic approach has much affinity with the way psychiatric disorders are classified: In the case of MDD, symptoms involve cognitive patterns (*e.g.* guilt or suicidal ideation), behaviors (*e.g.* inactivity or a suicide attempt), and physiological changes (*e.g.* losing weight). Taking the DSM-5 criteria (20), MDD could be expressed by 227 different variants of the symptoms, without even taking their severity into account (21). For attention-deficit-/hyperactivity disorder (ADHD), actually even more than 10,000 variations could be distinguished on the basis of the DSM criteria. This demonstrates a high degree of *descriptive heterogeneity* of such disorder categories. As Glannon described, Biological Psychiatry found neuroscientific patterns statistically correlated with such instances of MDD and many other disorders. But not a single one of the hundreds of disorders classified in the DSM can generally be described, let alone individually diagnosed, on the neuroscientific level alone (22, 23). Reductionism does not seem to be a successful paradigm in psychiatry, with clinical researchers looking for a brain-based nosology since more than 170 years (24), when psychiatric disorders are not generally classified on the neuroscientific level, cannot be diagnosed on that level in individual cases, and a patient’s treatment response cannot be assessed there alone. It has been discussed elsewhere that this can be partially explained by the limitations of present methodology (22, 25) or the normativity of psychiatric disorders (26, 27).

## Implications for Patients’ Treatment Choices

**Figure 1:**
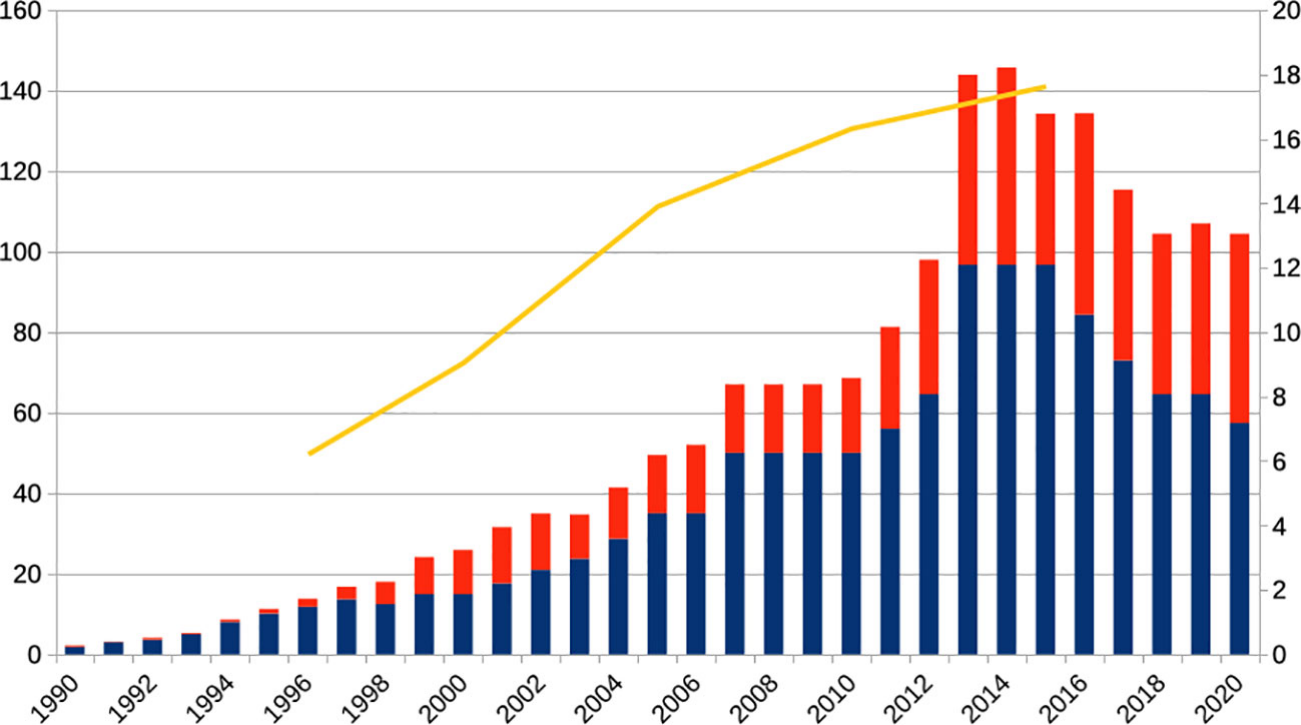
Annual production of the schedule II regulated ADHD drugs amphetamine (red) and methylphenidate (blue, left scale, x 1,000 kg) and adult patients annually receiving antidepressant drugs in the United States (yellow, right scale, x 1,000,000). The production of the ADHD drugs has increased more than thirtyfold, the number of MDD patients receiving antidepressants about threefold in the shown period. It must be also noted, though, that in the period shown the criteria for diagnosing ADHD changed, for example, with respect to *adult* ADHD and the DSM-5 of 2013 (20). Source: updated from (28), US Federal Register; (29).

So far, the discussion was primarily relevant to researchers and, to a lesser degree, clinicians. Glannon (1) raised concerns about mind–brain dualism in psychiatry and related patients’ understanding of psychiatric disorders to the risk that they might eschew effective biological treatments when they take a limited psychological stance. Besides the new approaches on brain stimulation described by Glannon, medical consumption patterns indicate, though, that ever more people are taking psychopharmacological drugs. For example, the *annual* production of ADHD medication in the US is presently higher than during the whole *decade* of the 1990s (28). The pattern for other psychopharmacological drugs (**Figure 1**) and in many other countries is similar (30).

Humans have actually used substances (*e.g.* alcohol or other stimulating, relaxing, or hallucinogenic drugs) instrumentally, for their psychological effects, probably as long as we exist (31, 32). The recent neuro-enhancement debate exemplifies a similar trend to improve one’s cognitive performance or feelings pharmacologically (28, 33). It thus seems to be common knowledge in human history that behavior and experience can be affected by consuming certain substances or performing particular rituals (*e.g.* dance, sports, or prayer). Whether or not many people have dualistic views, believing in the existence of independent “minds” or “souls”, they nevertheless seem to endorse biological means to solve their problems or achieve their aims, perhaps even increasingly so. Many decades ago, Klerman described that people differ in their readiness to take pharmacological drugs, distinguishing the extreme poles of “Psychotropic Hedonism” on the one hand and “Pharmacological Calvinism” on the other, but rather due to their lifestyle choices than philosophical world-views (34, 35). After the “Decade of the Brain” and the Human Genome Project, the analysis of science communication patterns and people’s descriptions of their psychiatric problems rather suggest, in contrast to Glannon’s concern, that patients might presently rather underestimate the value of *psychotherapy*, not biological treatments (36, 37).

## Conclusion: Integration Instead of Reduction

Variants of dualism have been discussed in philosophy for centuries. I tried to show here that we neither need to postulate “souls” nor “minds” to describe people’s behaviors or experiences. To overcome dualism, reifying “the mind” won’t help. Using a dualistic language that postulates “mind–brain interactions” reiterates old questions about the nature of the mental entity, its mechanism of interaction with the brain/body, and gives rise to endless discussions on reductionism that neither seems to be fruitful nor relevant to empirical research in psychology or the neurosciences. The biopsychosocial model and mechanistic explanations can take different levels of descriptions into account, without demanding reductions; as colleagues and I explained earlier, neuroscience can also be integrative (38). That people probably always have and are still using different means to change their brain/body and thus facilitate certain behaviors and experiences is also rather compatible with a pluralistic than a dualistic or reductionistic view. Instead of proposing an either-or-account, it goes without saying that many biological treatments have psychological effects (*i.e.* effects that we can only describe on the psychological level) and that psychological treatments are changing the brain/body. To ensure that psychiatric patients can consent to the best available treatment, it is essential, in my view, that they are informed in a neutral, pluralistic, and comprehensible way about all different options.

## Author Contributions

The author confirms being the sole contributor of this work and has approved it for publication.

## Funding

This publication has been supported by the “History of Neuroethics” grant by the Dutch Research Foundation (NWO), grant number 451-15-042.

## Conflict of Interest

The author declares that the research was conducted in the absence of any commercial or financial relationships that could be construed as a potential conflict of interest.

## Footnotes

1 The term *reification* is derived from the Latin *res*, thing. Reification means thus to consider something as a thing. Talking about brain–mind-interaction thus assumes that there are two entities, brain and mind, which are interacting in some way.
